# Deciphering the Therapeutic and Preventive Potential of Dietary Tannins in Osteosarcoma: A Multi‐Omics Approach Targeting TGFA and Immune Microenvironment Remodeling

**DOI:** 10.1002/fsn3.72041

**Published:** 2026-06-28

**Authors:** Ge Yunling, Xu Jietao, Xiao He, Zhao Tingxiao, Lv Jun, Zhou Hong, Qian Jiawei, Wang Xijun, Kang Yao

**Affiliations:** ^1^ Department of Orthopedics, Cancer Center Zhejiang Provincial People's Hospital, Affiliated People's Hospital, Hangzhou Medical College Hangzhou Zhejiang China; ^2^ Department of Laboratory Medicine Zhejiang Provincial People's Hospital, Affiliated People's Hospital, Hangzhou Medical College Hangzhou Zhejiang China; ^3^ Department of Dermatology Peking University Third Hospital Beijing China; ^4^ Department of Orthopedics, Center for Plastic & Reconstructive Surgery Zhejiang Provincial People's Hospital, Affiliated People's Hospital, Hangzhou Medical College Hangzhou Zhejiang China; ^5^ Department of Head and Neck Surgery Sun Yat‐Sen University Cancer Center Guangzhou China; ^6^ State Key Laboratory of Oncology in South China Guangzhou Guangdong China; ^7^ Collaborative Innovation Center for Cancer Medicine Guangzhou Guangdong China

**Keywords:** dietary polyphenol, machine learning, multi‐omics, osteosarcoma, tannin, TGFA

## Abstract

Malignant tumors, including osteosarcoma (OS), are major non‐communicable chronic diseases driven by systemic inflammation and oxidative stress. While dietary tannins possess known antioxidant and anticancer properties, their precise regulatory mechanisms and therapeutic targets in OS remain largely unexplored. This study integrated multi‐omics datasets to systematically investigate the potential of dietary tannins in OS, utilizing a machine‐learning framework based on 10 algorithms to construct a tannin‐related risk (TRR) score. The TRR model demonstrated favorable prognostic performance in retrospective cohorts, with high‐TRR patients exhibiting poorer survival, enrichment of extracellular matrix remodeling pathways, and reduced CD8^+^ T‐cell infiltration. Furthermore, immunotherapy prediction tools suggested a lower likelihood of response to immune checkpoint blockade in the high‐TRR group. TGFA was identified as a core hub gene contributing to the high‐risk phenotype; single‐cell and spatial transcriptomic analyses revealed that TGFA‐high OS cells exhibit stem‐like features and enhanced microenvironmental communication. In vitro assays confirmed that TGFA knockdown suppresses OS cell proliferation and migration while increasing apoptosis, whereas TGFA overexpression promotes these malignant behaviors. By bridging dietary polyphenol research with oncogenic management, this study identifies the TGFA‐associated immune axis as a precise molecular roadmap for the structural modification of polyphenols and the design of target‐specific, tannin‐based functional foods for chronic disease intervention.

## Graphical abstract

Dietary tannin‐associated molecular programs converge on a TGFA‐centered regulatory axis that governs osteosarcoma progression and immune remodeling. Multi‐omics integration combined with machine learning identifies a tannin‐related risk score (TRR) that stratifies tumor aggressiveness and immune landscape heterogeneity. TGFA‐high states promote tumor proliferation and suppress CD8^+^ T‐cell infiltration, whereas TGFA‐low states are associated with immune activation and improved prognosis, highlighting TGFA as a mechanistic bridge between dietary polyphenol exposure and tumor ecosystem reprogramming.

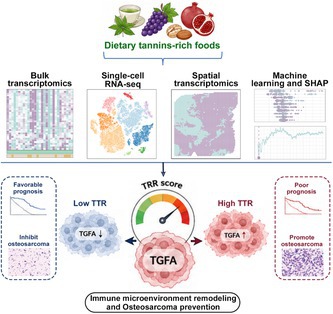

## 1 Introduction

Dietary polyphenols represent a diverse and extensive group of plant‐derived secondary metabolites commonly found in a wide array of human dietary sources, such as fruits, vegetables, tea, coffee, nuts, and whole grains (Ran et al. 2024). These bioactive compounds are well‐documented for their potent antioxidant and anti‐inflammatory properties, which play a fundamental role in providing protection against the development and progression of various chronic diseases (Rudrapal et al. 2024). Within the broad landscape of these phytochemicals, tannins have emerged as a distinct and structurally significant subclass, garnering increasing attention for their unique chemical nature and multifaceted biological activities (Molino et al. 2023; Sahakyan et al. 2020). Found in foods like tea, red wine, nuts, and legumes, tannins are primarily classified into hydrolyzable and condensed types, each exhibiting unique biological activities (Zayed et al. 2025; Cosme et al. 2025). Experimental and epidemiological studies have suggested that tannins may exert antioxidant, anti‐inflammatory, antimicrobial, and anticancer effects, supporting their potential relevance to chronic disease prevention and adjunctive cancer‐related applications (Baer‐Dubowska et al. 2020; Kleszcz et al. 2025). Concurrently, malignant tumors represent a significant category of non‐communicable chronic diseases (NCCDs) globally, characterized by complex pathologies driven by sustained inflammation and cellular aberrations. Given that dietary components are instrumental in systemic regulation, exploring the preventive and therapeutic potential of plant secondary metabolites like tannins against such chronic malignancies aligns with current paradigms in lifestyle‐based disease management (Fabbrini et al. 2022).

Osteosarcoma (OS) is the most prevalent primary malignant bone tumor, primarily affecting children, adolescents, and young adults (Beird et al. 2022). Despite significant advancements in surgical interventions and chemotherapy, the prognosis for OS remains dismal, particularly in cases involving metastatic disease (Gill and Gorlick 2021; Yu and Yao 2024). These challenges underscore the urgent need for improved therapeutic strategies and biomarkers to enhance early detection and treatment outcomes. While the potential of tannins in preventing chronic diseases has been increasingly recognized, their specific role in OS remains largely unexplored (Hoque et al. 2025). Given their antioxidant, anti‐inflammatory, and anti‐proliferative activities, tannin‐associated biological processes may provide useful clues for identifying molecular programs involved in OS progression and treatment response (Tan et al. 2025; Maugeri et al. 2022).

Unlike traditional structure–function studies or pharmacokinetic investigations, multi‐omics analyses provide an opportunity to explore disease‐relevant molecular programs associated with bioactive compounds at the systems level (Jiang et al. 2022; Zhang et al. 2026). This strategy is particularly useful when direct pharmacological interpretation is limited by complex metabolism, low systemic exposure, or incomplete mechanistic evidence. By integrating toxicogenomic resources with transcriptomic profiles, clinical outcomes, single‐cell states, and spatial organization, it is possible to identify tannin‐associated genes and pathways that are linked to OS progression and tumor microenvironment remodeling (Zheng, Liu, et al. 2024). Crucially, elucidating these explicit downstream genetic targets and microenvironmental profiles provides a vital molecular blueprint to overcome the inherent limitations of dietary polyphenols, such as poor stability and low bioavailability. Identifying such receptive pathways is foundational for directing future structural modifications of tannins and formulating targeted delivery systems, ultimately driving the rational design of polyphenol‐based functional foods optimized for chronic disease intervention.

In this study, we investigated the molecular relevance of tannin‐associated genes in OS using an integrated multi‐omics strategy. By combining multiple public datasets, we employed machine‐learning algorithms to construct a tannin‐related prognostic model and identify key molecular contributors associated with OS progression. We further used single‐cell RNA sequencing and spatial transcriptomics to characterize the cellular context, spatial distribution, and tumor microenvironmental features of these targets. Finally, cell‐based experiments were performed to validate the functional role of the leading candidate gene in regulating OS cell proliferation, migration, and apoptosis. This integrated approach provides new insights into tannin‐associated molecular programs in OS, highlighting potential biomarkers and therapeutic targets for further investigation. More broadly, by mapping these targeted genomic networks, this study framework supports the strategic development of personalized nutrition and targeted functional food interventions aimed at mitigating severe oncogenic NCCDs.

## 2 Methods

### 2.1 Data Acquisition and Processing

Transcriptomic expression profiles and corresponding clinical information for OS were obtained from the GEO and TARGET databases. mRNA expression matrices from all cohorts were collected, and genes with low expression levels (expression value ≤ 0.5) across samples were excluded to improve data reliability and reduce background noise. Clinical information was matched to the corresponding expression matrices, and samples with overall survival times < 30 days were removed from subsequent analyses. The GSE42352 dataset was used for differential expression analysis to identify tannin‐associated genes relevant to OS. The TARGET‐OS cohort served as the training dataset, whereas three GEO datasets (GSE16091, GSE21257, and GSE39055) were integrated as an external validation cohort. Because these datasets were generated from different platforms, batch effects were first evaluated using principal component analysis (PCA) and subsequently corrected using the ComBat algorithm (Johnson et al. 2007). The corrected datasets were then merged into a unified validation cohort for downstream analyses and model evaluation.

### 2.2 Identification of Tannin‐Associated Genes

Tannin‐associated genes were identified by querying the Comparative Toxicogenomics Database (CTD) with an Interaction Count > 1 to retrieve genes interacting with condensed or hydrolyzable tannins. In parallel, GeneCards was searched using a GIFtS score > 50 to identify genes with established associations. These high‐confidence genes were then integrated into a comprehensive panel for further downstream analysis.

### 2.3 Differential Expression and ssGSEA Analyses

Differential expression analysis was performed to evaluate transcriptional alterations in tannin‐associated genes in OS samples. Comparisons were conducted between predefined sample groups according to the corresponding study design, and genes with a nominal *p* < 0.05 were considered differentially expressed. In addition, single‐sample gene set enrichment analysis (ssGSEA) was used to quantify the enrichment score of the tannin‐associated gene set in each sample. These scores were subsequently used to assess the association between tannin‐related gene activity and the clinical or biological features of OS.

### 2.4 Machine Learning‐Based Model Construction

Several machine‐learning algorithms were applied to construct prognostic models, including Random Survival Forests (RSF), Lasso‐Cox, CoxBoost, Gradient Boosting Machine (GBM), plsRcox, and stepwise Cox regression. All models were trained using the same tannin‐associated gene set, and predictive performance was compared using the concordance index (*C*‐index) in both the training and validation cohorts.

### 2.5 RSF Model Establishment and Performance Assessment

The RSF model was further optimized using out‐of‐bag (OOB) error estimation. Genes contributing to survival prediction were ranked according to variable importance scores. Patients were stratified into high‐ and low‐risk groups based on RSF‐derived Tannin‐Related Risk Scores (TRR) in both the training and validation cohorts. Model performance was evaluated using Kaplan–Meier survival analysis and time‐dependent receiver operating characteristic (ROC) curves.

### 2.6 SHAP‐Driven Interpretation of Gene Contributions

SHapley Additive exPlanations (SHAP) analysis was performed to interpret the contribution of individual genes to the RSF model. Mean absolute SHAP values were calculated to quantify the overall importance of each tannin‐associated gene. SHAP summary plots were generated to visualize both the magnitude and direction of feature effects on the RSF‐derived risk score. SHAP dependence plots were then used to assess the relationship between gene expression levels and their corresponding effects on model output, allowing visualization of nonlinear associations and potential feature interactions. In addition, SHAP force/waterfall plots were generated to decompose individualized predictions and illustrate how selected genes contributed to the predicted risk score for each patient.

### 2.7 Functional Annotation of Cohort‐Consistent Differentially Expressed Genes

Differential expression analysis was performed separately in the TARGET‐OS training cohort and the merged validation cohort. Genes with |log_2_FC| > 1 and *p* < 0.05 were defined as differentially expressed in the training cohort, whereas genes with |log_2_FC| > 0.1 and *p* < 0.05 were identified in the validation cohort. Upregulated and downregulated genes identified from both cohorts were intersected to obtain consistently altered gene sets. These concordant upregulated and downregulated genes were subsequently subjected to functional enrichment analysis. Kyoto Encyclopedia of Genes and Genomes (KEGG) enrichment was conducted to identify overrepresented signaling pathways, and Gene Ontology (GO) enrichment analysis was performed across biological process, cellular component, and molecular function categories.

### 2.8 Assessment of Immune Infiltration

To compare immune cell composition between the high‐ and low‐TRR groups, four transcriptome‐based deconvolution algorithms, including CIBERSORT (Newman et al. 2015), MCPcounter (Becht et al. 2016), EPIC (Racle et al. 2017), and quanTIseq (Finotello et al. 2019), were applied to estimate immune cell abundance. The resulting immune infiltration profiles were compared between TRR‐defined subgroups. For each algorithm, Pearson correlation analysis was performed to evaluate the association between TGFA expression and estimated CD8^+^ T‐cell abundance.

### 2.9 Prediction of Immunotherapy Response

To assess potential sensitivity to immune checkpoint blockade, Tumor Immune Dysfunction and Exclusion (TIDE) scores were calculated for each sample (Jiang et al. 2018), and the predicted responder proportions were compared between the high‐ and low‐TRR groups. Immunophenoscore (IPS) analysis was used to estimate tumor immunogenicity based on antigen presentation, effector cell activity, and immune checkpoint‐related features. In addition, SubMap analysis was performed to compare TRR‐defined subgroups with reference immunotherapy response signatures, providing an independent prediction of potential treatment sensitivity (Zheng, Hai, et al. 2024).

### 2.10 Preprocessing and Integration of Single‐Cell Data

Single‐cell transcriptomic profiles from the OS dataset GSE162454 were analyzed using Seurat (v5.3.0). Cells exhibiting excessive mitochondrial transcription (> 20%) or insufficient gene complexity (< 300 or > 6000 detected genes) were discarded to ensure data quality. Following log‐normalization, variable genes were selected for downstream modeling, and principal component analysis was used to capture major transcriptional variations. Clustering was performed using a shared nearest neighbor‐based approach, and Harmony integration was applied to mitigate inter‐sample variability. Two‐dimensional embeddings were generated with t‐SNE for visualization, and cellular identities were assigned based on well‐established lineage markers. To further characterize tumor cell populations, TGFA expression levels were assessed to define transcriptionally distinct OS cell subsets. CytoTRACE was used to estimate differentiation status, and CellChat was employed to infer putative ligand‐receptor signaling between OS cells and other microenvironmental components.

### 2.11 Spatial Transcriptomic Analysis

Spatial transcriptomic data from the SP_BS3 OS specimen were processed using Seurat (v5.3.0). After quality filtering, SCTransform was applied for normalization, followed by PCA and UMAP for dimensionality reduction and spatial clustering. To evaluate the activity of the tannin‐related molecular program at spatial resolution, we calculated “tannin‐related scores” for each spot using the AUCell algorithm. Cell identities were assigned based on canonical marker expression, and tissue domains were delineated using spatial pattern‐based inference. To estimate the cellular composition of each spot, scRNA‐seq profiles from GSE162454 were integrated through SPOTlight (v1.2.0), enabling deconvolution of major tumor and microenvironmental populations. The spatial distribution of OS cells, TGFA‐high and TGFA‐low subgroups, and immune cell subsets was visualized using scatterpie‐based mapping, and regional differences in TGFA expression and cell‐state enrichment were subsequently quantified.

### 2.12 Cell Culture

U2OS, MG‐63, and HEK‐293T cell lines were obtained from Procell (Wuhan, China). U2OS and MG‐63 OS cell lines were maintained in McCoy's 5A medium (Gibco, USA) and EMEM (Gibco, USA), respectively, while HEK‐293 T cells were cultured in DMEM (Gibco, USA). All media were supplemented with 10% fetal bovine serum (Gibco, USA) and 1% penicillin–streptomycin (Yeasen, China). Cells were incubated at 37°C in a humidified atmosphere containing 5% CO_2_ and passaged every 2–3 days at 80%–90% confluence. All cell lines used in this study were routinely tested and confirmed to be free of mycoplasma contamination, and their identities were verified by the supplier prior to use.

### 2.13 Cell Transfection

shRNA plasmids targeting TGFA and cDNA plasmids encoding TGFA were purchased from Addgene (Beijing Zhongyuan Co., China). HEK‐293T cells were used for lentiviral packaging. U2OS and MG‐63 cells at approximately 70% confluence were transduced with lentiviral particles in the presence of polybrene (Shanghai GeneChem, China). Forty‐eight hours after transduction, cells were collected for subsequent experiments or subjected to puromycin selection for 2 weeks to establish stable cell lines. TGFA knockdown and overexpression efficiencies were confirmed by quantitative real‐time PCR (qRT‐PCR). The sequences used for TGFA silencing and overexpression are listed in Table S1.

### 2.14 RNA Extraction and Quantitative Real‐Time PCR

Total RNA was extracted from U2OS or MG‐63 cells using TRIzol reagent (Invitrogen, USA). RNA concentration and purity were assessed using a NanoDrop 2000 spectrophotometer (Thermo Fisher Scientific, USA). cDNA was synthesized using the PrimeScript RT kit (Takara, Japan) according to the manufacturer's instructions. qRT‐PCR was performed using Power SYBR Green Master Mix (Yeasen, China). Each sample was analyzed in triplicate, and GAPDH was used as the internal control. Relative expression levels were calculated using the 2^−^ΔΔCt method. Primer sequences are provided in Table S2.

### 2.15 Cell Proliferation Assays

Cell proliferation was assessed using CCK‐8 and EdU assays. For CCK‐8 detection, cells were seeded into 96‐well plates (5 × 10^3^ cells/well), incubated for 24–72 h, treated with 10 μL CCK‐8 reagent (Yeasen, China) for 2 h at 37°C, and absorbance was measured at 450 nm. EdU incorporation was evaluated using a commercial kit (RiboBio, China); transfected cells were exposed to 50 μM EdU for 3 h, fixed, stained with Apollo dye and Hoechst, and imaged using a fluorescence microscope (Olympus, Japan).

### 2.16 Cell Migration Assay

Cell migration was examined using Transwell chambers with 8‐μm pores (Corning, USA). A total of 5 × 10^4^ cells suspended in serum‐free medium were seeded into the upper chamber, whereas the lower chamber was filled with medium containing 10% FBS. After 24 h, migrated cells were fixed with 4% paraformaldehyde, stained with 0.1% crystal violet, imaged, and counted.

### 2.17 Apoptosis Assay

Apoptosis was measured using an Annexin V‐FITC/PI kit (KeyGen Biotech, China) according to the manufacturer's protocol. The apoptosis rate was calculated as the sum of early apoptotic cells (Annexin V^+^/PI^−^) and late apoptotic cells (Annexin V^+^/PI^+^).

## 3 Results

### 3.1 Expression Landscape of Tannin‐Associated Genes in OS

**FIGURE 1:**
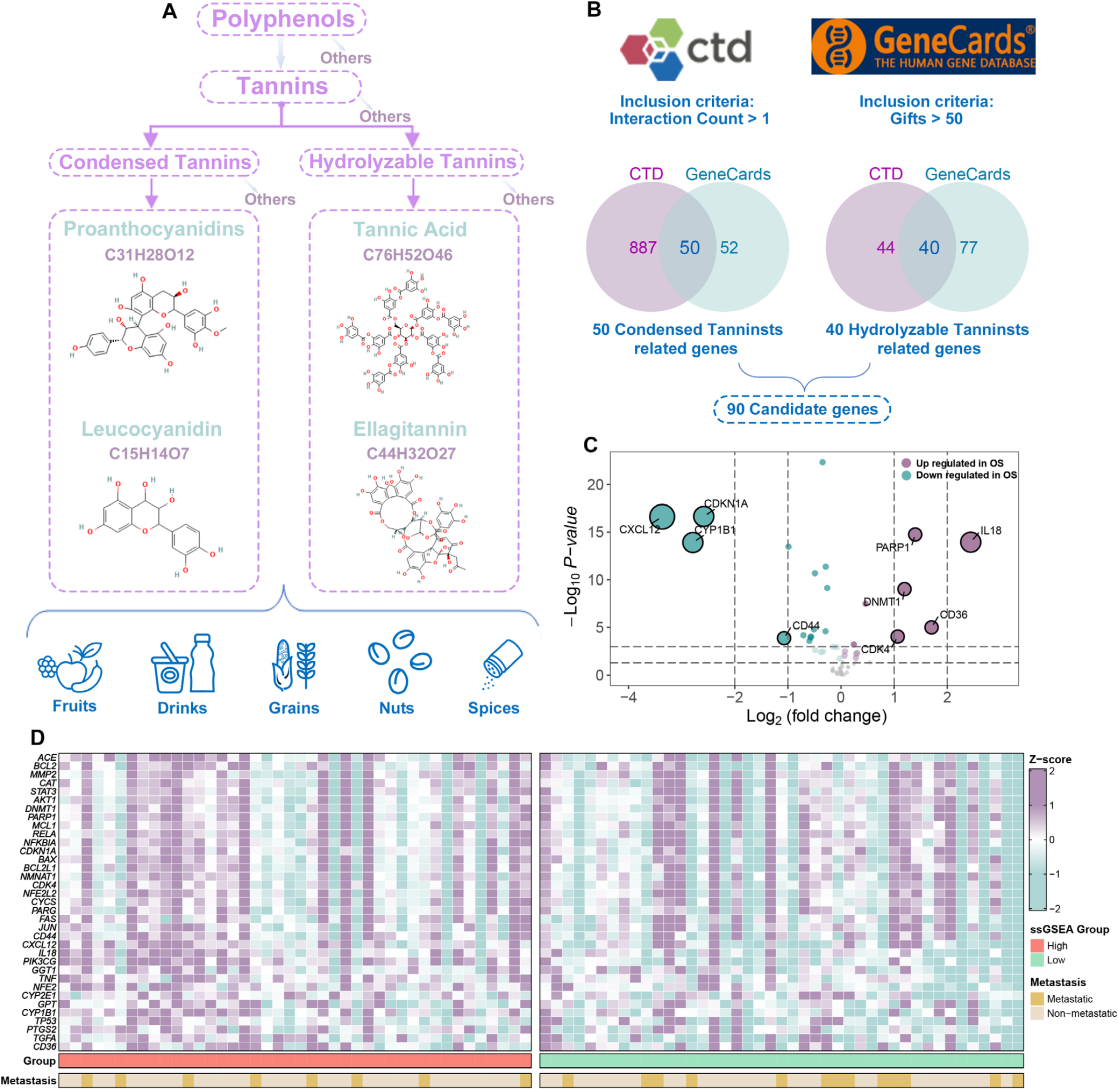
Overview of tannin subclasses and transcriptomic analysis of tannin‐associated genes in OS. (A) Classification of tannins, including condensed and hydrolyzable tannins, with representative molecular structures. (B) Integration of tannin‐associated genes obtained from the Comparative Toxicogenomics Database (CTD) using Interaction Count > 1 and from GeneCards using GIFtS score > 50. (C) Volcano plot showing differential expression of tannin‐associated genes based on the GSE42352 dataset. A total of 35 genes met the significance threshold (*p* < 0.05) and were classified into upregulated or downregulated groups. (D) Heatmap illustrating the expression patterns of the 35 differentially expressed tannin‐associated genes across OS samples, annotated by ssGSEA‐derived grouping and metastatic status.

To explore the potential relevance of tannin‐associated genes in OS, we first summarized representative tannin compounds, including condensed and hydrolyzable tannins, as shown in Figure 1A. Tannin‐associated genes were then collected from CTD and GeneCards, and overlapping genes identified from both databases were retained as high‐confidence candidates for subsequent transcriptomic analysis (Figure 1B). Using the GSE42352 dataset, differential expression analysis identified 35 tannin‐related genes that met the predefined significance threshold (*p* < 0.05). The distribution of these significantly altered genes is depicted in the volcano plot, which highlights distinct upregulated and downregulated transcripts within OS samples (Figure 1C). Further visualization of their expression profiles revealed transcriptional heterogeneity across the cohort, with distinct expression patterns related to ssGSEA‐derived grouping and metastatic status (Figure 1D). Collectively, these findings indicate that a subset of tannin‐associated genes is dysregulated in OS and may be associated with tumor heterogeneity and metastatic features.

### 3.2 Multi‐Cohort Development and Evaluation of an RSF‐Based Prognostic Framework

**FIGURE 2:**
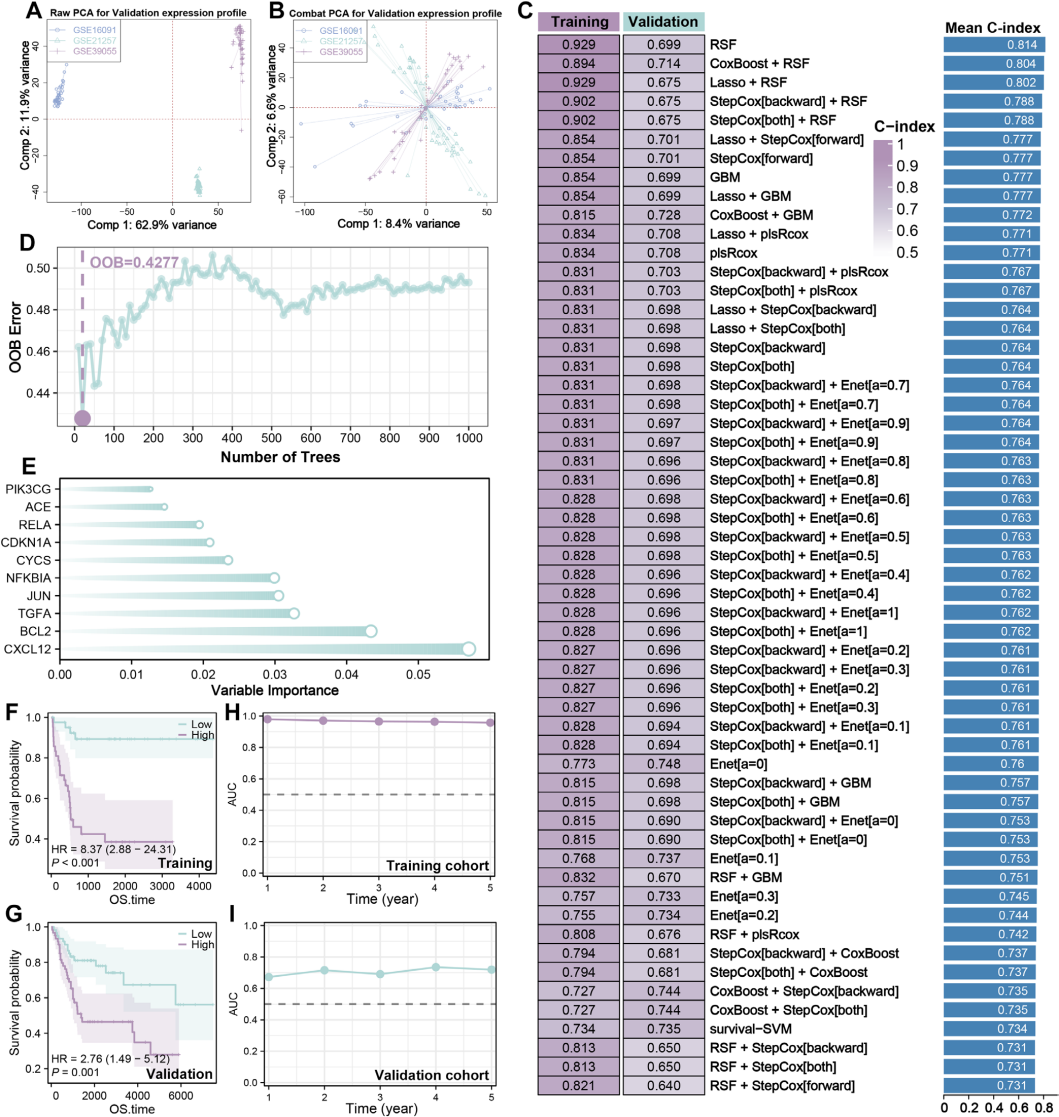
Multi‐cohort analysis of RSF‐derived prognostic models in osteosarcoma. (A) Principal component analysis (PCA) of the external validation datasets (GSE16091, GSE21257, GSE39055) before batch correction. (B) PCA of the combined validation cohort after batch effect correction using the Combat algorithm, showing reduced batch effects. (C) Performance comparison of several survival models, including Random Survival Forests (RSF), Lasso‐Cox, CoxBoost, Gradient Boosting Machine (GBM), plsRcox, and stepwise Cox regression. The RSF model achieved the highest concordance index (*C*‐index = 0.814). (D) Out‐of‐bag (OOB) error curve for the RSF model, demonstrating model stability as the number of trees increased. (E) Variable importance analysis of the RSF model, showing the top 10 genes contributing to survival prediction. (F, G) Kaplan–Meier survival curves for high‐ and low‐TRR groups in the training cohort (F) and the external validation cohort (G), both showing significant survival separation. (H, I) Time‐dependent receiver operating characteristic (ROC) curves evaluating the predictive accuracy of the RSF model in the training cohort (H) and the validation cohort (I).

Using the 35 differentially expressed tannin‐associated genes identified from the GSE42352 dataset, we constructed a prognostic framework for OS. The TARGET‐OS cohort served as the training dataset, while GSE16091, GSE21257, and GSE39055 were incorporated as external validation datasets. Principal component analysis revealed clear separation among the raw datasets, whereas batch effects were markedly reduced following Combat correction, enabling integration of the validation datasets into a unified cohort (Figure 2A,B). To identify an appropriate predictive approach, multiple machine‐learning algorithms were systematically compared. Among them, the Random Survival Forests (RSF) model achieved the highest concordance index and was therefore selected for subsequent analysis (Figure 2C). The RSF model was established using the 35‐gene signature, and the OOB error curve indicated stable model performance (Figure 2D). Variable importance analysis highlighted the top 10 genes that contributed most prominently to the RSF predictive system (Figure 2E). Based on RSF‐derived TRR scores, patients in the TARGET‐OS cohort were stratified into high‐ and low‐TRR groups, which showed significantly different survival outcomes (Figure 2F). Consistent TRR stratification performance was observed in the external validation cohort, further supporting the robustness of the TRR‐based classification (Figure 2G). Time‐dependent receiver operating characteristic curves further showed favorable predictive performance in both the training and validation cohorts (Figure 2H,I). These findings suggest that the RSF‐derived TRR may serve as a prognostic indicator for OS, although further validation in prospective or real‐world cohorts is warranted.

### 3.3 Elucidating Feature Contributions to RSF Predictions Using SHAP Analysis

**FIGURE 3:**
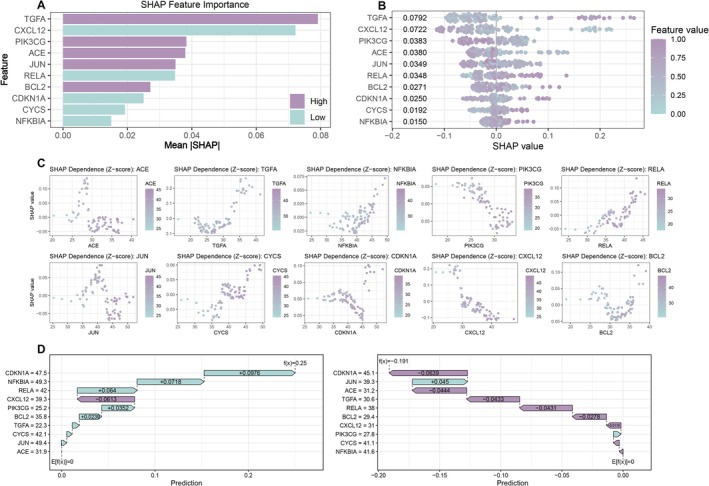
SHAP‐based interpretation of gene contributions to the RSF prognostic model. (A) SHAP feature importance ranking showing the average absolute contribution of each tannin‐associated gene to the TRR. (B) SHAP summary plot illustrating both the magnitude and direction of each gene's effect on the predicted risk. (C) SHAP dependence plots depicting the relationship between gene expression values and corresponding SHAP contributions for the top features. (D) SHAP force (waterfall) plots demonstrating the individualized contribution of key genes to the predicted risk score for representative patients.

To interpret the contribution of individual genes to the TRR, SHAP analysis was performed. SHAP feature importance ranking showed that TGFA, CXCL12, and PIK3CG had the largest contributions to the predictive output, followed by ACE, JUN, RELA, BCL2, CDKN1A, CYCS, and NFKBIA (Figure 3A). The SHAP summary plot further illustrated both the magnitude and direction of each gene's effect on the TRR, indicating that higher expression of genes such as TGFA and RELA was associated with increased predicted risk, whereas higher expression of genes such as CXCL12 and CYCS was associated with reduced predicted risk (Figure 3B). SHAP dependence plots showed nonlinear relationships between gene expression levels and corresponding SHAP values, highlighting gene‐specific contribution patterns and potential feature interactions among the top‐ranked genes, including ACE, TGFA, NFKBIA, PIK3CG, RELA, JUN, CYCS, CDKN1A, CXCL12, and BCL2 (Figure 3C). To further examine individualized predictions, SHAP force plots were used to visualize how selected genes contributed additively to the predicted risk scores of representative high‐ and low‐risk patients (Figure 3D). Together, these findings provide an interpretable view of gene‐level contributions within the RSF model and identify candidate genes potentially associated with TRR‐defined risk stratification.

### 3.4 Functional Divergence Between Consistently Upregulated and Downregulated Genes

**FIGURE 4:**
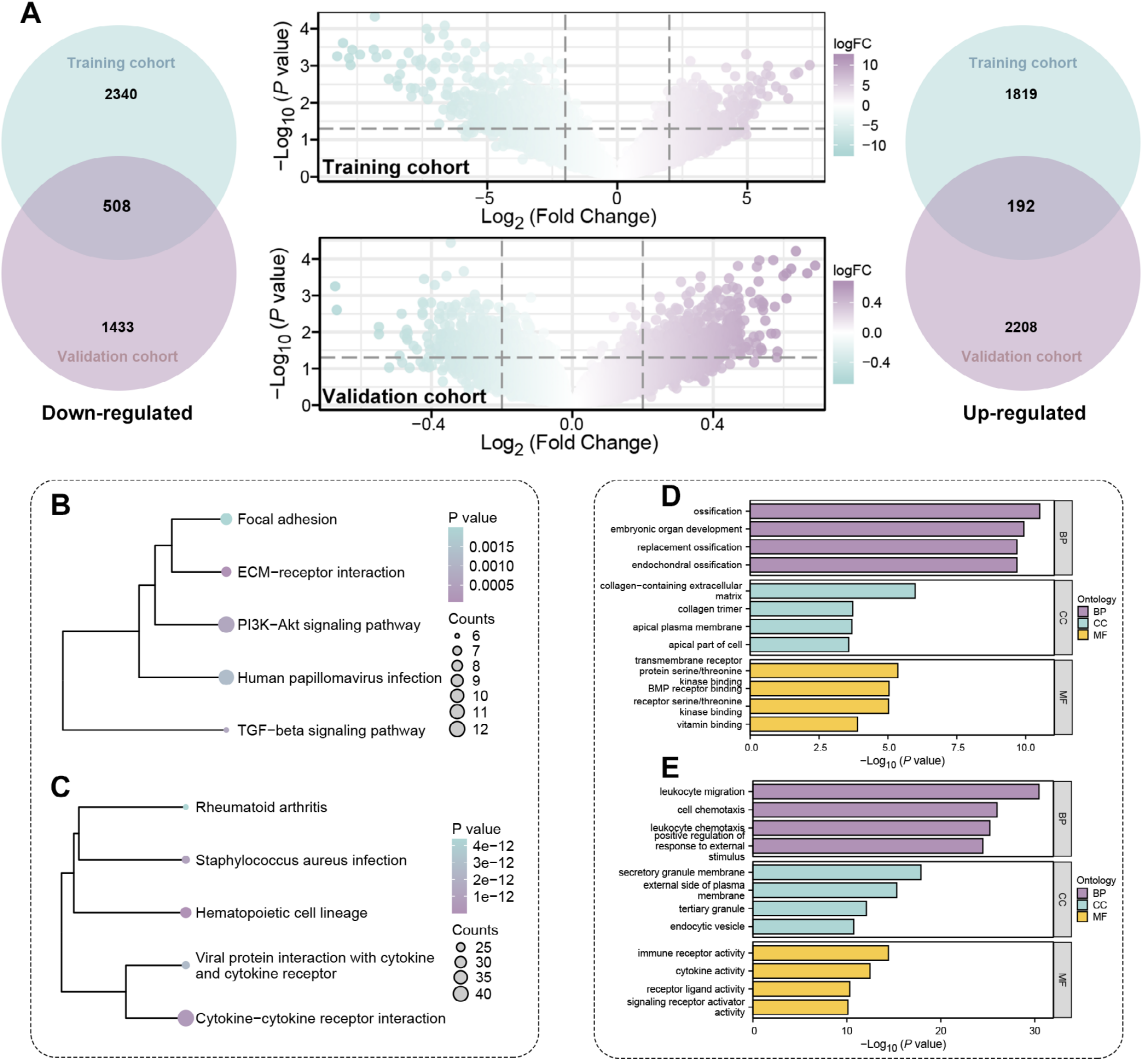
Functional divergence between cohort‐consistent differentially expressed genes. (A) Identification of consistently upregulated and downregulated genes across the TARGET‐OS training cohort and the merged validation cohort. (B) KEGG enrichment analysis of consistently upregulated genes, showing significant enrichment in matrix‐ and signaling‐related pathways. (C) KEGG enrichment analysis of consistently downregulated genes, highlighting immune‐ and inflammation‐associated pathways. (D) GO enrichment analysis of consistently upregulated genes across biological process, cellular component, and molecular function categories. (E) GO enrichment analysis of consistently downregulated genes, indicating enrichment of immune activation and chemotaxis‐related terms.

Differential expression analysis was performed in the TARGET‐OS training cohort and in the merged validation cohort, and genes with matching upregulation or downregulation patterns across datasets were used for functional enrichment (Figure 4A). KEGG enrichment analysis revealed that the upregulated subset was predominantly associated with tumor‐promoting pathways, including focal adhesion, ECM‐receptor interaction, PI3K‐Akt signaling, TGF‐β signaling, and related matrix remodeling processes (Figure 4B). In contrast, the consistently downregulated subset was enriched for immune‐ and inflammation‐related pathways, such as cytokine‐cytokine receptor interaction, hematopoietic cell lineage, and multiple infection‐ and immune‐response pathways (Figure 4C). GO enrichment further highlighted this functional divergence, showing that upregulated genes were enriched in biological processes related to ossification, embryonic organ development, and extracellular matrix organization, together with membrane‐associated cellular components and receptor‐binding molecular functions (Figure 4D). Conversely, downregulated genes mapped to leukocyte migration, chemotaxis, immune activation, and secretory granule‐associated components, accompanied by receptor and cytokine activity at the molecular function level (Figure 4E). Together, the data reveal a coordinated pattern in which upregulated genes cluster in matrix and developmental pathways, whereas downregulated genes are predominantly enriched in immune‐related processes.

### 3.5 Immune Landscape and Immunotherapy Response Patterns Associated With TRR

**FIGURE 5:**
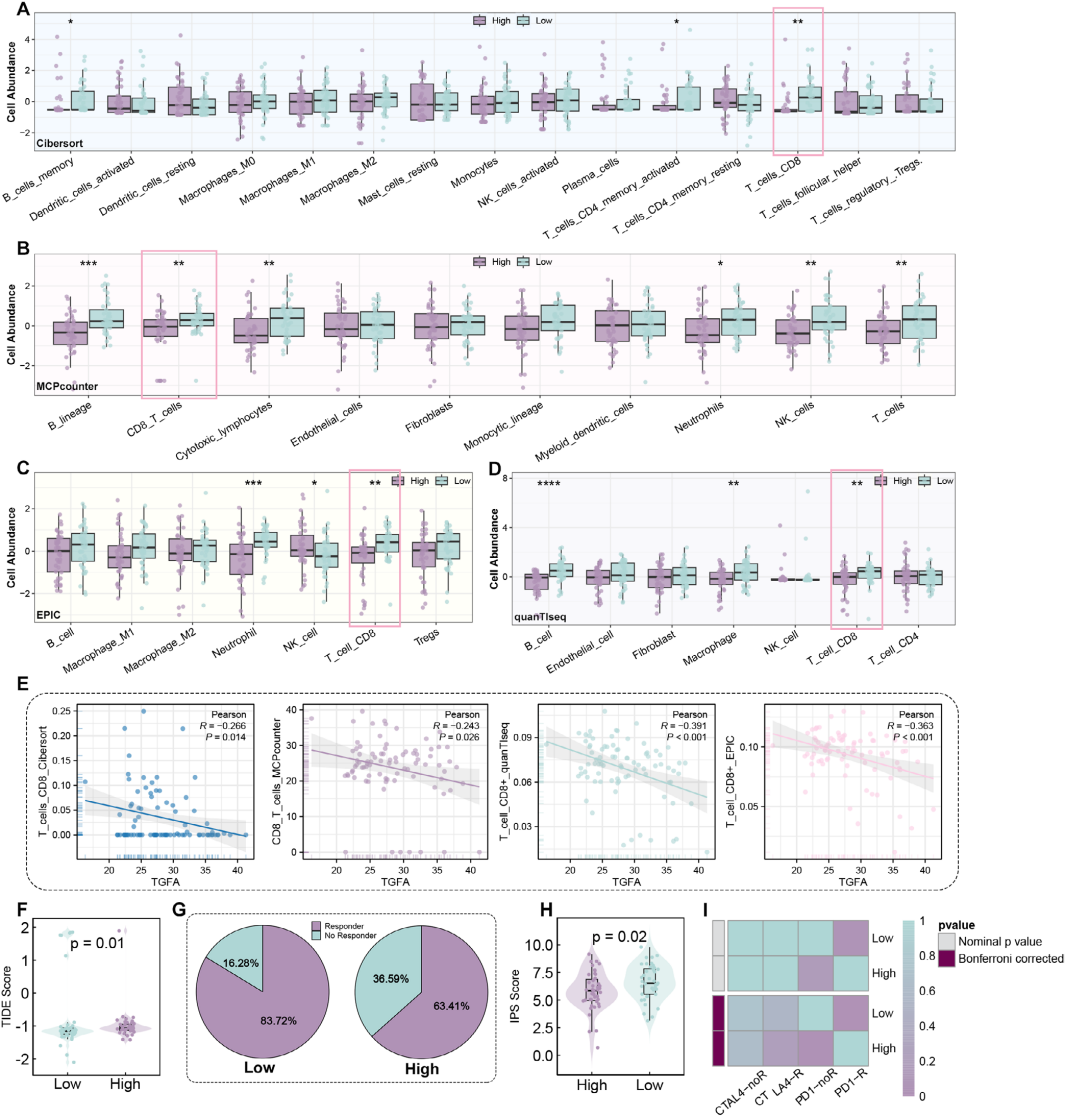
Immune infiltration and predicted immunotherapy response across TRR subgroups. (A–D) Comparison of immune cell infiltration between high‐ and low‐TRR groups using four deconvolution algorithms (CIBERSORT, MCPcounter, EPIC, and quanTIseq). CD8^+^ T‐cell abundance showed consistent differences across methods. (E) Correlation analysis between TGFA expression and CD8^+^ T‐cell infiltration across multiple algorithms. (F, G) TIDE analysis showing TIDE score and predicted immunotherapy responder proportions in TRR‐defined subgroups. (H) Immunophenoscore (IPS) results comparing predicted immunogenicity between groups. (I) SubMap analysis evaluating predicted responsiveness to immune checkpoint blockade. *p < 0.05, **p < 0.01, ***p < 0.001, ****p < 0.0001.

Multiple deconvolution algorithms were applied to compare the immune infiltration profiles between high‐ and low‐TRR groups, including CIBERSORT, MCPcounter, EPIC, and quanTIseq. Across all four methods, most immune cell populations showed no substantial differences between groups; however, CD8^+^ T‐cell abundance consistently differed and was significantly lower in the high‐TRR group in every algorithm (Figure 5A–D). Given that TGFA was identified as the most influential gene in the SHAP analysis, its association with CD8^+^ T‐cell infiltration was further evaluated. TGFA expression showed a statistically significant, albeit modest, negative correlation with CD8^+^ T‐cell abundance across all computational frameworks (R values ranging from approximately −0.24 to −0.39), suggesting a consistent, yet complex, relationship between TGFA and immune exclusion (Figure 5E). To assess predicted immunotherapy responsiveness, TIDE analysis revealed lower TIDE scores in the low‐TRR group, suggesting reduced immune evasion potential (Figure 5F), and a higher proportion of predicted responders to immune checkpoint blockade (Figure 5G). Consistently, immunophenoscore (IPS) analysis demonstrated significantly higher IPS values in the low‐TRR group (Figure 5H), and SubMap analysis further indicated a stronger predicted response to immunotherapy within this group (Figure 5I). Collectively, these findings suggest that although global immune infiltration patterns are broadly similar between TRR‐defined subgroups, CD8^+^ T‐cell activity is selectively diminished in the high‐TRR group, accompanied by multiple lines of evidence pointing to superior immunotherapy sensitivity in patients with low TRR.

### 3.6 Single‐Cell Characterization of TGFA‐Associated OS Cell States

**FIGURE 6:**
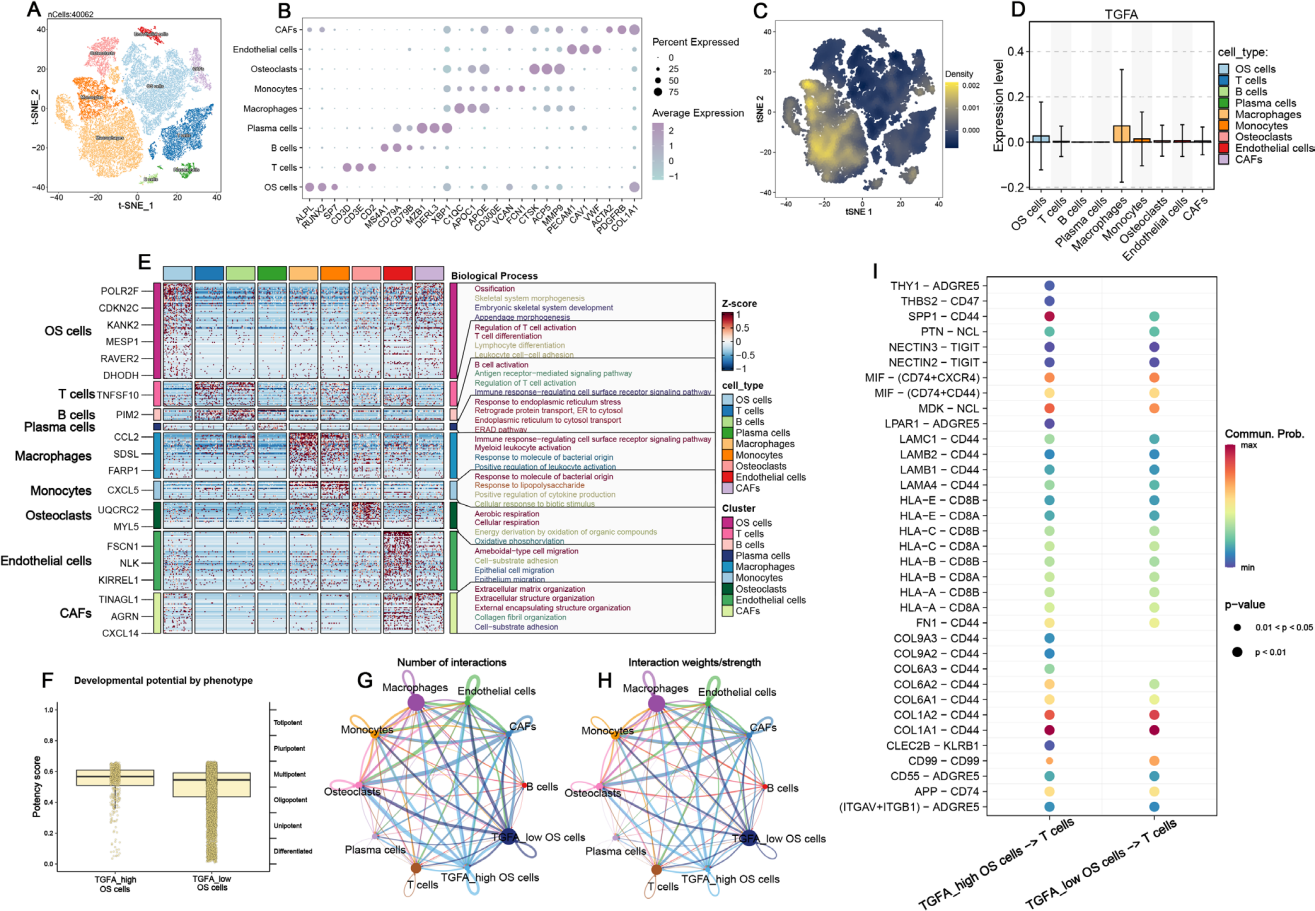
Single‐cell characterization of TGFA‐associated osteosarcoma cell states. (A) t‐SNE plot showing major cell populations identified in the GSE162454 single‐cell dataset. (B) Dot plot displaying the expression of canonical marker genes across annotated cell types. (C) Density ma*p*of single cells projected onto the t‐SNE embedding. (D) TGFA expression levels in different cell types. (E) Heatma*p*of representative marker genes for each cluster and the corresponding enriched GO biological processes. (F) Comparison of inferred developmental potential between TGFA‐high and TGFA‐low OS cells. (G) Cell–cell communication network depicting the number of inferred ligand‐receptor interactions among major cell populations, including TGFA‐high and TGFA‐low OS cells. (H) Network showing the overall interaction strength between the same cell populations. (I) Dot plot of selected ligand‐receptor pairs between OS cells and T cells, highlighting stronger immunosuppressive T cell‐related interactions in TGFA‐high OS cells than in TGFA‐low OS cells.

To characterize the cellular context of TGFA in OS, we analyzed the single‐cell dataset GSE162454 and identified major tumor and microenvironmental populations, including OS cells, immune cell subsets, endothelial cells, osteoclasts, plasma cells, and CAFs, each occupying distinct transcriptional clusters (Figure 6A,B). TGFA expression was mainly detected in macrophages and OS cells and showed marked intratumoral heterogeneity (Figure 6C,D). Based on TGFA expression levels, OS cells were stratified into TGFA‐high and TGFA‐low groups. TGFA‐high OS cells showed higher inferred developmental potential and a less differentiated transcriptional state (Figure 6E,F). Cell–cell communication analysis suggested that TGFA‐high OS cells had more extensive and stronger predicted ligand‐receptor interactions with surrounding immune and stromal compartments (Figure 6G,H). Several interactions enriched in TGFA‐high OS cells were related to T‐cell inhibitory or dysfunctional states, suggesting a potential association between TGFA‐high tumor cells and immunosuppressive signaling features (Figure 6I). Collectively, these findings indicate that TGFA‐high OS cells may represent a stem‐like, transcriptionally active subpopulation with enhanced microenvironmental communication and possible involvement in immunosuppressive signaling in OS.

### 3.7 Spatial Distribution of TGFA‐Associated OS Cell States

**FIGURE 7:**
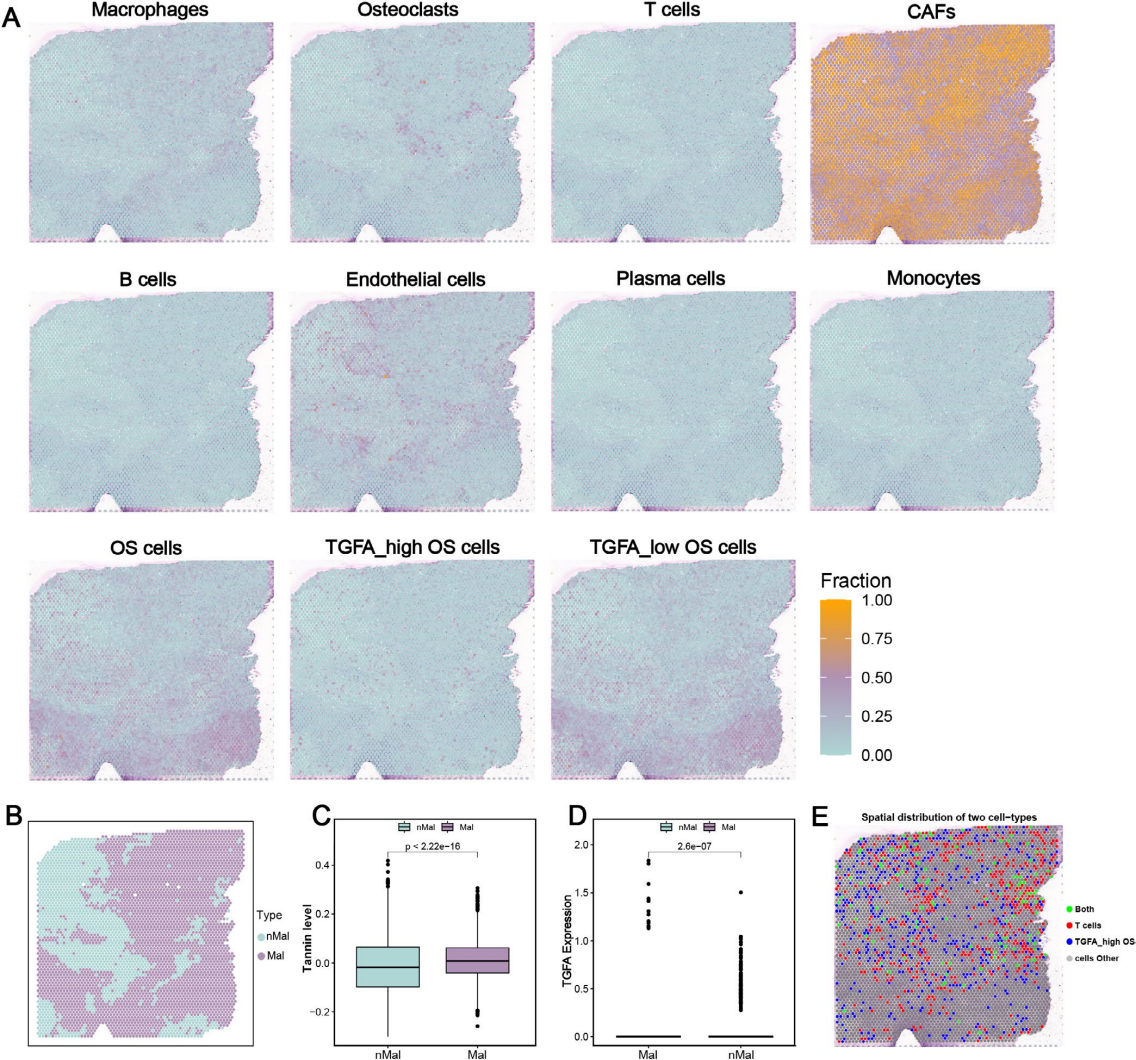
Spatial transcriptomic analysis of TGFA‐associated osteosarcoma cell states. (A) Spatial distribution of major OS and microenvironmental cell populations inferred from deconvolution of the SP_BS3 spatial transcriptomic sample. (B) Spatial segmentation of malignant (Mal) and nonmalignant (nMal) tissue regions. (C) Comparison of tannin‐related scores between malignant and nonmalignant regions. (D) TGFA expression levels in malignant versus nonmalignant regions. (E) Spatial co‐localization of TGFA‐high OS cells and T cells, indicating spots containing either cell type or both.

Spatial transcriptomic profiling of the SP_BS3 sample revealed distinct spatial patterns of major tumor and microenvironmental cell populations. Macrophages, osteoclasts, T cells, B cells, endothelial cells, plasma cells, monocytes, CAFs, and OS cells exhibited heterogeneous distributions across the tissue section, with CAFs showing prominent regional enrichment (Figure 7A). Spatial domain segmentation further separated malignant and nonmalignant regions (Figure 7B), within which malignant areas demonstrated significantly higher tannin‐related scores and elevated TGFA expression (Figure 7C,D). Co‐localization analysis demonstrated that TGFA‐high OS cells tended to appear in spots also containing T cells, indicating a spatial proximity and potential interaction between these populations (Figure 7E). These findings suggest a spatial association between TGFA‐high OS cells and T cells in the analyzed tissue section, which is consistent with the single‐cell communication analysis. However, because the spatial transcriptomic analysis was based on one available OS specimen, these results should be interpreted as exploratory and hypothesis‐generating rather than definitive evidence of a generalizable spatial pattern.

### 3.8 TGFA Promotes OS Cell Proliferation, Migration, and Survival

**FIGURE 8:**
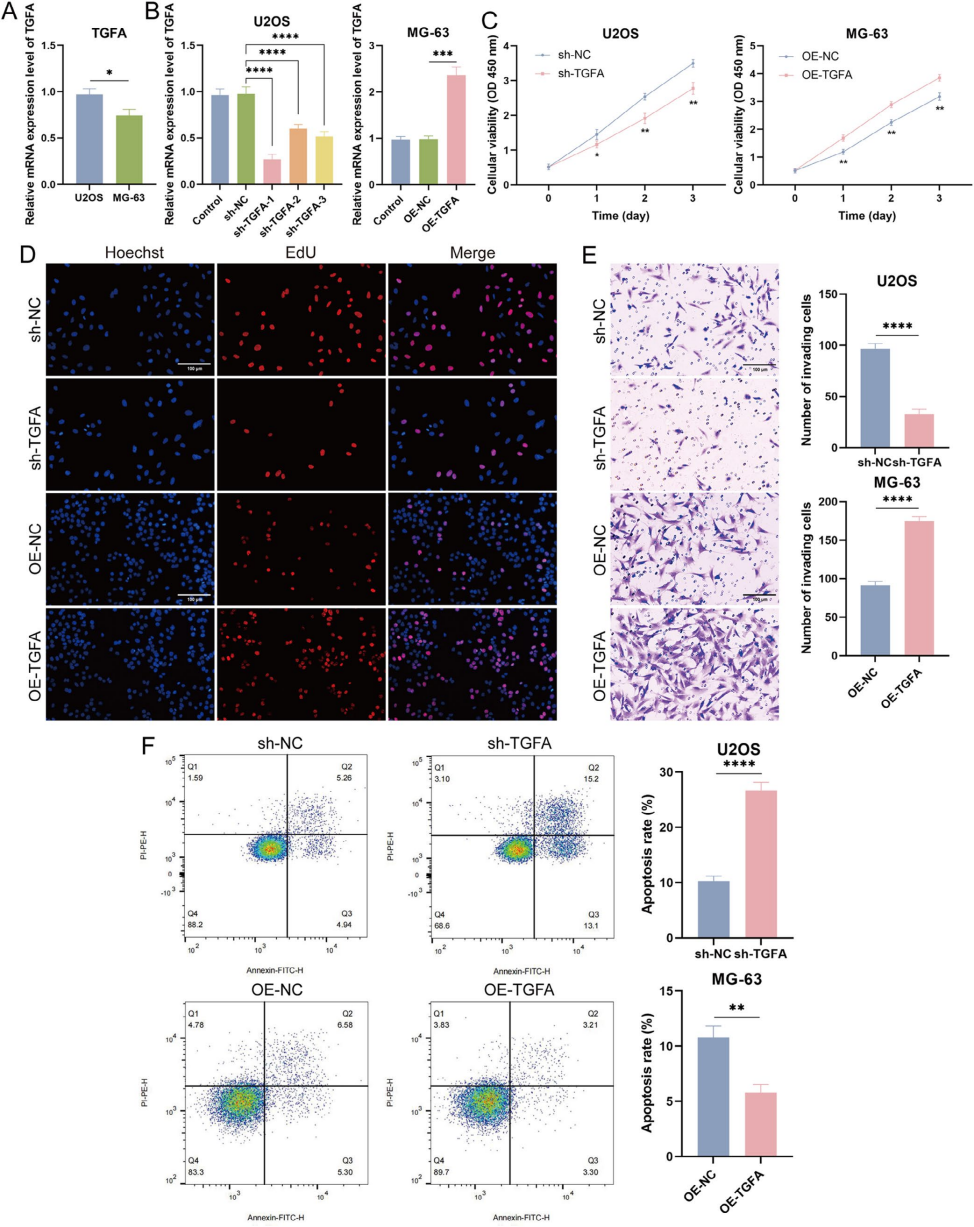
Functional validation of TGFA in osteosarcoma cells. (A) Basal TGFA mRNA expression levels in U2OS and MG‐63 cells. (B) Verification of TGFA knockdown in U2OS cells and overexpression in MG‐63 cells by qRT‐PCR. (C) CCK‐8 assays showing the effects of TGFA knockdown and overexpression on cell viability. (D) EdU staining indicating changes in proliferative capacity following TGFA knockdown or overexpression. (E) Transwell assays evaluating the impact of TGFA on osteosarcoma cell migration. (F) Flow cytometry analysis of apoptosis in TGFA‐silenced U2OS cells and TGFA‐overexpressing MG‐63 cells.*p < 0.05, **p < 0.01, ***p < 0.001, ****p < 0.0001.

The baseline expression of TGFA differed between OS cell lines, with MG‐63 cells exhibiting higher endogenous TGFA levels than U2OS cells (Figure 8A). Efficient TGFA knockdown and overexpression were confirmed in U2OS and MG‐63 cells, respectively (Figure 8B). Functional assays showed that TGFA silencing significantly reduced cell viability and EdU incorporation in U2OS cells, whereas TGFA overexpression enhanced these proliferative capacities in MG‐63 cells (Figure 8C,D). Consistently, Transwell assays revealed that TGFA knockdown markedly impaired cell migration, while TGFA overexpression promoted invasive behavior (Figure 8E). Flow cytometry further demonstrated that TGFA depletion increased apoptosis, whereas TGFA overexpression decreased apoptotic rates in OS cells (Figure 8F). These results collectively indicate that TGFA enhances proliferative and migratory abilities while conferring survival advantages to OS cells.

## 4 Discussion

In this study, we systematically integrated multi‐database gene mining, multi‐cohort transcriptomic modeling, single‐cell and spatial analyses, and functional experiments to explore the biological and clinical relevance of tannin‐associated genes in OS. Clinically, malignant tumors such as OS represent a highly severe and aggressive category of global NCCDs, which are driven by sustained systemic inflammation, immune evasion, and oxidative stress. While epidemiological evidence widely acknowledges the general health benefits of dietary polyphenol intake, traditional empirical studies often lack systemic, target‐specific molecular resolution. Our findings provide several critical observations that bridge the ga*p*between dietary polyphenol research and oncogenic NCCD management. First, we identified a distinct subset of tannin‐related genes that were dysregulated in OS, suggesting that tannin‐associated molecular programs may be linked to OS‐related biological processes beyond their traditional antioxidant and anti‐inflammatory roles (Baer‐Dubowska et al. 2020; Youness et al. 2021; Rajasekar et al. 2021). Second, based on these genes, we developed a Random Survival Forest–based TRR score, which demonstrated favorable prognostic performance across retrospective public datasets. By delivering a robust machine learning‐derived TRR framework, our study transitions the understanding of dietary tannins from non‐specific phytochemicals to a quantifiable, target‐specific network capable of dictating the microenvironmental landscape of complex chronic malignancies.

The TRR framework further unveiled biologically distinct OS phenotypes, providing mechanistically rich insights for targeted nutrition. High‐TRR tumors were enriched for extracellular matrix remodeling, PI3K‐Akt, and TGF‐β signaling, pathways previously implicated in tumor invasion and metastatic progression in OS (Zhou et al. 2025; Choi et al. 2024). Conversely, genes downregulated in high‐risk patterns were mainly enriched in immune‐related processes, suggesting that reduced immune activity may be associated with the high‐TRR phenotype. Among the immune features evaluated, CD8^+^ T‐cell abundance was consistently lower in high‐TRR samples across four deconvolution algorithms. This finding suggests a potential association between high TRR and a less cytotoxic immune microenvironment, which may partly contribute to poorer outcomes in these patients. In addition, multiple immunotherapy prediction tools, including TIDE, IPS, and SubMap, suggested that low‐TRR patients may have a higher likelihood of response to immune checkpoint blockade. Crucially, mapping these explicit downstream genetic pathways offers a unique molecular framework to overcome the inherent limitations of dietary polyphenols, which frequently exhibit low target‐tissue accumulation when administered in their native form.

To achieve higher resolution, we employed single‐cell and spatial transcriptomics to dissect the cellular and spatial distribution of tannin‐associated programs within the OS ecosystem. Single‐cell RNA‐seq profiling identified TGFA as a highly prominent hub gene within the tannin‐associated regulatory network. In our spatial analysis of the representative specimen SP_BS3, TGFA transcripts localized to malignant mesenchymal cell niches and areas characterized by high extracellular matrix remodeling. Furthermore, TGFA expression correlated inversely with CD8^+^ T‐cell localization, which aligns with previous reports linking the ligand TGFA to impaired antitumor immunity through the hyperactivation of EGFR signaling (Yarden and Sliwkowski 2001). Our cell‐based experiments provided functional validation of these multi‐omics insights, showing that silencing TGFA reduced OS cell proliferation and migration while increasing apoptosis. These results suggest that TGFA may act as a downstream regulator in the tannin‐regulatory framework, orchestrating both aggressive cell phenotypes and an immunosuppressive microenvironment.

Importantly, from a translational and food sciences perspective, these pharmacokinetic insights directly address the critical bottleneck of dietary polyphenols highlighted in current NCCD research. Tannins are well known to face extensive gastrointestinal transformation and low systemic bioavailability, as they are rapidly metabolized by the gut microbiota or restricted by limited intestinal absorption. The discovery of TGFA as a pivotal, receptive downstream target within our tannin‐regulatory network provides a critical molecular roadma*p*to address this challenge. Future bioengineering and food technology efforts can leverage this specific target to guide the structural modification of plant‐derived tannins—such as targeted esterification or polymerization—or to design sophisticated polyphenol‐protein delivery complexes and nano‐formulations. By customizing these formulations to specifically target the hyper‐activated TGFA/EGFR axis and reverse the immunosuppressive microenvironment characterized in our study, researchers can significantly enhance the stability, tissue‐specific accumulation, and functional bioavailability of tannins, ultimately driving the rational design of polyphenol‐based functional foods optimized for chronic malignancy intervention (Ciardiello and Tortora 2008; Singh et al. 2016).

Despite the strengths of this multi‐modal approach, several critical limitations warrant consideration. First, our spatial transcriptomic insights were derived from a single representative OS specimen (SP_BS3); therefore, the generalizability of these spatial patterns to a broader patient population remains to be validated in larger cohorts. Second, our functional validation focused solely on TGFA knockdown and overexpression rather than direct treatment with dietary tannin compounds, leaving the precise biochemical link between tannins and TGFA expression to be established. Most importantly, the absence of an immunocompetent in vivo model means that our claims regarding the TGFA‐driven immune landscape remain primarily predictive. Future functional and clinical studies are warranted to validate these therapeutic hypotheses using living immune systems and controlled pharmacological dosing.

In conclusion, this study uncovers a previously unrecognized link between tannin‐related molecular programs and OS biology, contextualized within the broader framework of NCCD chemical prevention. The TRR provides a robust tool for prognostic stratification, while TGFA is identified as a putative regulator of an aggressive OS cell state and an unsupportive immune microenvironment. More broadly, by integrating advanced multi‐omics technologies (toxicogenomics, single‐cell RNA‐seq, and spatial transcriptomics), our workflow exemplifies a systematic approach to explore the dee*p*anti‐NCCD mechanisms of dietary polyphenols. These findings lay a solid scientific foundation for personalized nutrition and targeted dietary interventions, moving a ste*p*closer to managing severe NCCDs through engineered functional foods.

## Author Contributions

**Xiao He:** conceptualization, writing – original draft, investigation, methodology, visualization, validation, writing – review and editing, software, formal analysis, data curation, resources. **Ge Yunling:** conceptualization, methodology, software, data curation, formal analysis, validation, investigation, visualization, resources, writing – original draft, writing – review and editing. **Zhao Tingxiao:** writing – original draft, writing – review and editing. **Qian Jiawei:** investigation, formal analysis, software. **Lv Jun:** visualization, validation. **Zhou Hong:** data curation, resources. **Wang Xijun:** funding acquisition, project administration, supervision. **Kang Yao:** project administration, funding acquisition, supervision. **Xu Jietao:** conceptualization, investigation, writing – original draft, methodology, validation, visualization, writing – review and editing, software, formal analysis, data curation, resources.

## Funding

This research was supported by grants from the Zhejiang Provincial Natural Science Foundation of China (grant no. LQ24H160045) and the Zhejiang Provincial Health Bureau Science Foundation of China (grant no. 2024KY647).

## Ethics Statement

The authors have nothing to report.

## Consent

The authors have nothing to report.

## Conflicts of Interest

The authors declare no conflicts of interest.

## Supporting information


**Table S1:** The sequences for TGFA knockdown and overexpression.

**Table S2:** Primers for qRT‐PCR.

## Data Availability

All datasets used in this study are described in detail in the Methods section. Additional information and materials supporting the findings of this work are available from the corresponding author upon reasonable request.
