# Supplementary material for: Deciphering the Therapeutic and Preventive Potential of Dietary Tannins in Osteosarcoma: A Multi‐Omics Approach Targeting TGFA and Immune Microenvironment Remodeling
Source: Food Sci Nutr. 2026 Jun 28;14(7):e72041. doi: 10.1002/fsn3.72041 (PMC13310960; doi:10.1002/fsn3.72041)
Supplement: Supplementary file 1 — Table S1: The sequences for TGFA knockdown and overexpression. [file FSN3-14-e72041-s001.docx]

**Table S1.** The sequences for TGFA knockdown and overexpression.

| Name | sense 5’-3’ | antisense 5’-3’ |
| --- | --- | --- |
| sh-TGFA-1 | CCGGAGTGTGACCTAGAGAAGAAATCTCGAGATTTCTTCTCTAGGTCACACTTTTTT | AATTAAAAAAGTGTGACCTAGAGAAGAAATCTCGAGATTTCTTCTCTAGGTCACACT |
| sh-TGFA-2 | CCGGCGGTAAGTATGTTTAGAAATACTCGAGTATTTCTAAACATACTTACCGTTTTT | AATTAAAAACGGTAAGTATGTTTAGAAATACTCGAGTATTTCTAAACATACTTACCG |
| sh-TGFA-3 | CCGGGGAGATTTCTATAGTTATTGTCTCGAGACAATAACTATAGAAATCTCCTTTTT | AATTAAAAAGGAGATTTCTATAGTTATTGTCTCGAGACAATAACTATAGAAATCTCC |
| OE-TGFA | AATGTACAAGGAATTCGCCACCATGGTCCCCTCGGCTGGAC | GAATTATCTAGGGATCCTCAGACCACTGTTTCTGAG |
