# Supplementary material for: Deciphering the Therapeutic and Preventive Potential of Dietary Tannins in Osteosarcoma: A Multi‐Omics Approach Targeting TGFA and Immune Microenvironment Remodeling
Source: Food Sci Nutr. 2026 Jun 28;14(7):e72041. doi: 10.1002/fsn3.72041 (PMC13310960; doi:10.1002/fsn3.72041)
Supplement: Supplementary file 2 — Table S2: Primers for qRT‐PCR. [file FSN3-14-e72041-s002.docx]

**Table S2.** Primers for qRT-PCR.

| Gene | Forward | Reverse |
| --- | --- | --- |
| TGFA | AGGTCCGAAAACACTGTGAGT | AGCAAGCGGTTCTTCCCTTC |
| GAPDH | GGAGCGAGATCCCTCCAAAAT | GGCTGTTGTCATACTTCTCATGG |
